# Effects of Black Garlic Polyphenols on the Physicochemical Characteristics, Antioxidant Activity, and Sensory Evaluation of Yogurt

**DOI:** 10.3390/gels11070561

**Published:** 2025-07-21

**Authors:** Weiwei Cao, Linlin Li, Jiancheng Wang, Weihua Guo, Wei Chen, Lifeng Pan, Duo Li

**Affiliations:** 1 Institute of Nutrition & Health, Qingdao University, 308 Ningxia Road, Qingdao 266071, China; caoweiwei@haust.edu.cn (W.C.); linlinli2020@126.com (L.L.); 2 College of Food and Bioengineering, Henan University of Science and Technology, Luoyang 471023, China; 3 R&D Center, Haitong Ninghai Foods Co., Ltd., Ninghai 315709, China; w_jc@haitonggroup.com (J.W.); gwh@haitonggroup.com (W.G.); chenwei@haitonggroup.com (W.C.); plf@haitonggroup.com (L.P.)

**Keywords:** black garlic polyphenols, yogurt, physicochemical characteristics, antioxidant activity

## Abstract

Yogurt fortified with polyphenols, as a new type of functional yogurt, exhibits high quality and good antioxidant activity. However, the effects of black garlic polyphenols (BGP) on the quality of solidified yogurt have been scarcely reported. Therefore, the effects of different levels (0.1–0.4%) of BGP on the sensory scores, physicochemical properties, rheological properties, texture properties, antioxidant activity and polyphenol content of solidified yogurt were studied. The results showed that the total sensory scores of yogurt with 0.2% and 0.4% levels of BGP addition were both above 80. BGP addition significantly decreased the water-holding capacity and pH values of yogurt, compared with the yogurt without BGP addition. The yogurt with a 0.4% level of BGP had the highest titratable acidity of 89.84° T. In addition, the storage modulus (G’) and loss modulus (G”) of yogurt increased with the addition of BGP. The gel strength, chewiness and hardness of yogurt significantly decreased with an increase in BGP addition. The content of quercetin and caffeic acid in the yogurt with the addition of 0.1–0.4% BGP was 0.53–1.79 mg/g and 2.13–4.98 mg/g, respectively. The antioxidant activity and total phenolic acid content of yogurt significantly increased with an increment in BGP addition. The 2,2′-azino-bis(3-ethylbenzothiazoline-6-sulfonic acid) diammonium salt radical scavenging activity, ferric reducing antioxidant power and 1,1-diphenyl-2-picrylhydrazyl radical scavenging activity of yogurt with a 0.4% level of BGP increased by 52.79%, 57.31% and 42.51%, respectively, compared to the yogurt without BGP addition. This study provides a theoretical basis for the development of novel yogurt with high antioxidant activity.

## 1. Introduction

Yogurt is a dairy product that is rich in proteins and probiotics and is popular among consumers of all age groups due to its pleasant taste and flavor. With the growing consumer demand resulting from the nutritional benefits of yogurt in the market, bioactive compounds such as polyphenols and phenolic acid extracts have been selected as supplements in yogurt to enhance the quality and nutritional value [1]. Polyphenols are widely distributed in fruit, vegetables and tea, exhibiting various health-beneficial functions, and are often adopted as ingredients to enhance food nutrition [2]. Rashwan et al. found that *Melastoma dodecandrum Lour* fruit powder significantly enhanced the anthocyanin content, proanthocyanidin content and antioxidant activity of yogurt [3]. Elderberry extracts were shown to increase the antioxidant activity of yogurt by 2.3 times due to its rich phenolic acids, compared with yogurt without the addition of elderberry extracts [4]. Apple pomace, rich in polyphenols, was shown to improve the elasticity and hardness of yogurt [5]. A previous study also showed that the water-holding capacity (WHC), texture and antioxidant activity of yogurt were improved by the addition of *Berberis aristata* fruit extract [6]. Hesperidin addition was proven to decrease the melting rate of frozen yogurt and increase yogurt hardness [7]. Therefore, phenolic acid extracts, as optimal additives, have the potential to be applied in developing novel yogurt with high biological activity and quality attributes.

Black garlic, as a type of fermented garlic, is a functional product derived from fresh garlic via prolonged high-temperature fermentation; it displays good antioxidant, anti-inflammatory and cardioprotective activity [8,9]. The high-temperature and high-humidity fermentation process induces the Maillard reaction, leading to the thermal degradation of garlic polysaccharides and a notable increase in the content of reducing sugars. The phenolic acid content in black garlic was found to be higher than that of fresh garlic due to heat processing under controlled humidity [8]. The phenolic acids in raw garlic primarily include gallic acid and caffeic acid, and these phenolic acids in processed black garlic showed different levels at different processing stages [10,11]. Black garlic extracts have been widely applied in the food industry to improve food quality. For example, the addition of black garlic extracts significantly inhibited the lipid oxidation of burgers and jerked beef meat with pork [12,13]. Black garlic in a semi-dry fermented sausage enhanced the content of esters, sulfur compounds and aldehydes [14]. BG has also been used to flavor chicken, fish, soup and risotto [15]. Although Shin et al. prepared yogurt with black garlic extracts (BGE) with lower phenolic acid content, only the basic physicochemical indicators were studied [16]. However, studies on the phenolic acid content and antioxidant activity of yogurt with higher content of black garlic polyphenol (BGP) extracts are scarcely reported. Therefore, this study investigated the effects of BGP addition on yogurt quality, including its sensory evaluation, physicochemical characteristics, rheological properties, texture, phenolic acid content and antioxidant properties.

## 2. Results and Discussion

### 2.1. Sensory Scores of Yogurt

**Figure 1:**
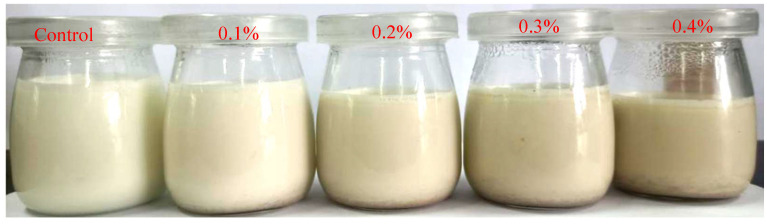
The yogurt with different levels of BGP.

The sensory evaluation results of yogurt with different levels of BGP are shown in Table 1. The figures of yogurt with different levels of BGP are shown in Figure 1. The yogurt without BGP exhibited the best flavor, taste and total score (91.00), which was significantly higher than that of the yogurt with BGP addition, suggesting that BGP had a slightly negative impact on the sensory properties of yogurt. However, Shin et al. found that yogurt with 0.5% BGE showed no significant differences compared to yogurt without BGE [16]. As BGP have higher phenolic acid content than BGE, the effects of BGP and BGE on the yogurt texture were different. Alamoudi et al. reported that 3% and 5% levels of phenolic acid extracts from mandarin pomace could decrease the overall acceptability score of yogurt [17]. The sensory scores of the yogurt with 0.1–0.4% BGP addition ranged from 70.67 to 84.00. The sensory scores of the yogurt with 0.2% and 0.3% levels of BGP were significantly higher than those of the yogurt with 0.1% and 0.4% levels of BGP. However, there were no significant differences between the sensory scores of the yogurt with a 0.2% level of BGP and a 0.3% level of BGP. The yogurt with a 0.4% level of BGP displayed the lowest scores for flavor, texture, taste and color, implying that excess BGP could negatively affect the sensory scores of yogurt. A 2.50–5.0% level of apple pomace syrup also decreased the consumer acceptance of Greek yogurt [18]. Images of yogurt with different levels of BGP are displayed in Figure 1. As the addition level of BGP increased, the color of the yogurt became darker, and the amount of whey separation in the yogurt increased. As excessive BGP imparted a strong garlic flavor and dark color to the yogurt, BGP significantly reduced the overall sensory acceptability of the yogurt. Therefore, based on the sensory evaluation and the antioxidant activity of yogurt, a 0.3% level of BGP was selected as the optimal addition level.

### 2.2. WHC of Yogurt

**Figure 2:**
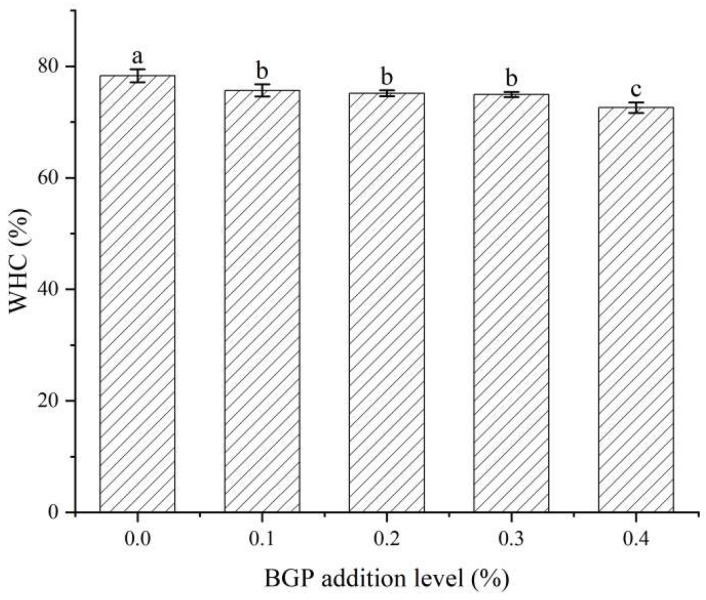
The WHC of yogurt with different levels of BGP. Different letters indicate significant differences (*p* < 0.05) (*n* = 3).

WHC as an important physicochemical indicator of yogurt quality could affect the texture stability of yogurt. Figure 2 shows the effects of different addition levels of BGP on the WHC of yogurt. The WHC values of the yogurt with 0%, 0.1%, 0.2%, 0.3% and 0.4% BGP were 78.30%, 75.67%, 75.15%, 74.94% and 72.59%, respectively. All levels of BGP decreased the WHC of yogurt. The yogurt without the addition of BGP had the highest WHC, and there were no significant differences in the WHC of the yogurt with 0.1–0.3% BGP. Compared with yogurt alone, the WHC of yogurt with 0.4% BGP decreased by 7.29%. This might be due to the fact that the formation of non-covalent bonds between milk proteins and BGP reduced the solubility of milk proteins [19]. Osorio-Arias et al. [20] reported that the addition of cheese whey and phenolic acid-rich coffee powder reduced the WHC of yogurt. Similarly, Golmakani et al. [21] found that the addition of pomegranate juice into yogurt led to a reduction in its WHC. Therefore, the addition level of BGP should not exceed 0.3% in order to maintain the optimal WHC of yogurt.

### 2.3. Titratable Acidity and pH of Yogurt

**Figure 3:**
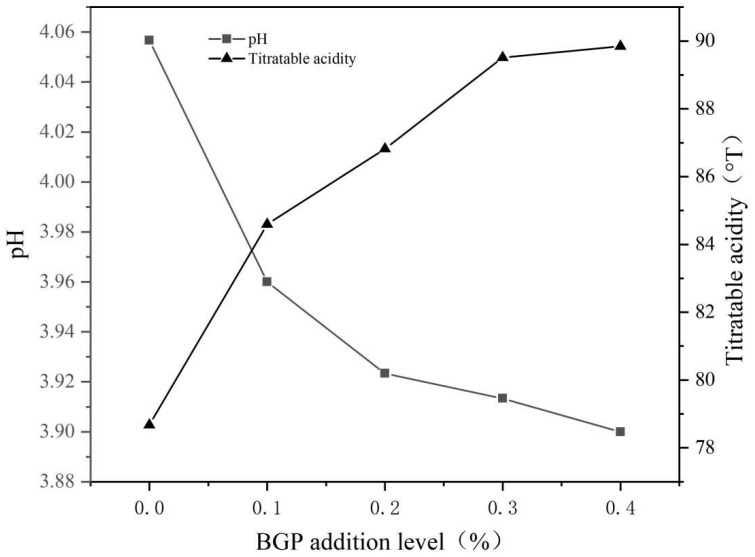
The pH value and titratable acidity of yogurt with different levels of BGP (*n* = 3).

The effects of different addition levels of BGP on the titratable acidity and pH of yogurt are shown in Figure 3. As the addition level of BGP increased, the pH of the yogurt decreased in a dose–effect relationship. The pH of the yogurt with 0–0.4% of BGP decreased from 4.06 to 3.96, while the titratable acidity increased from 78.68° T to 89.84° T. The decrease in pH and increase in titratable acidity might be attributed to the production of lactic acid by lactic acid bacteria during fermentation. Additionally, BGP might contribute to the growth of lactic acid bacteria and increase the production of lactic acid to induce an increase in the titratable acidity of yogurt [22,23]. This is in accordance with the fact that the pH of yogurt also decreased with increasing levels of bilberry pomace power [24].

### 2.4. Rheological Properties of Yogurt

**Figure 4:**
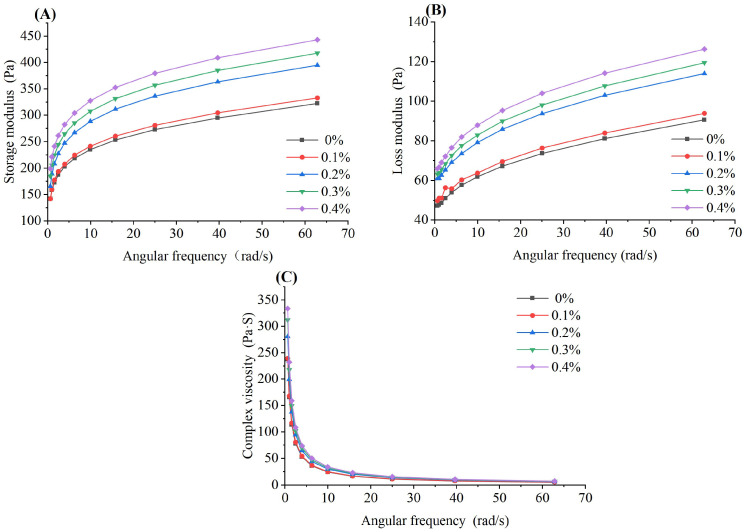
The rheological properties of yogurt with different levels of BGP. (**A**) Storage modulus, (**B**) loss modulus, (**C**) complex viscosity (*n* = 3).

The rheological properties of the yogurt with different levels of BGP are shown in Figure 4. As shown in Figure 4, the storage modulus (G’) and loss modulus (G”) of all yogurts increased with increasing shear frequencies. G’ and G” reflect the elastic and viscous properties of a material, respectively. The G’ values of all yogurts with different levels of BGP were higher than those of G”, indicating that the elastic properties of the yogurt were stronger than its viscous properties. Therefore, BGP addition did not change the weak semi-solid gel characteristics of the yogurt. Additionally, both the G’ and G” of yogurt were positively correlated with BGP addition and were higher than those of the yogurt without BGP addition, suggesting that BGP addition enhanced the elasticity and firmness of the yogurt. The phenomenon might be due to the fact that the interactions between polyphenols and proteins promoted stronger protein aggregation and stabilized the rigid gel structure of the yogurt. Bilberry pomace powder addition also increased the G’ and G” of yogurt [24]. However, the G’ and G” of yogurt were decreased by the addition of mulberry pomace. This might be due to the fact that the polyphenols in mulberry pomace are different from the polyphenols in BGP, which might bring about different effects on the milk protein interaction [25]. Figure 4C shows that the complex viscosity of the yogurt with different levels of BGP decreased with increasing angular frequencies, which was in agreement with the complex viscosity of yogurt fortified with air-dried apple pomace [5]. The complex viscosity at the same angular frequency increased with BGP addition, which was consistent with the viscosity tendency of yogurt containing mulberry pomace [25].

### 2.5. Texture Properties of Yogurt

The effects of different addition levels of BGP on the texture properties of yogurt are shown in Table 2. The gel strength, rupture strength, hardness, chewiness and stickiness of the yogurt with a 0.1% level of BGP were higher than those of yogurt alone and the yogurt with 0.2–0.4% of BGP, which might have been due to the formation of protein–polyphenol complexes, which improved the texture of the yogurt. The gel strength, rupture strength, hardness and chewiness of the yogurt significantly decreased with an increasing addition level of BGP, which was consistent with the tendency of the WHC of the yogurt with BGP. As the WHC of the yogurt decreased due to BGP, the protein–protein interactions and the original gel structure in the yogurt might have been weakened. However, the stickiness of yogurt with a 0.2–0.4% level of BGP had no significant discrepancy, which might be attributed to the fact that BGP did not affect the aggregation state of milk proteins and the interaction between proteins and milk fat. The gel strength, rupture strength and hardness of yogurt with a 0.4% level of BGP decreased by 13.31%, 8.42% and 8.42%, respectively, compared to the yogurt without BGP addition. As the formation of protein–polyphenol non-covalent complexes increased the particle sizes of milk proteins and prevented the cross-linking of casein, the gel strength and hardness of the yogurt decreased [26,27]. A previous study also reported that the hardness of yogurt was decreased with higher levels of *B. aristata* fruit extract [6].

### 2.6. Content of TPC, Quercetin and Caffeic Acid in Yogurt

**Figure 5:**
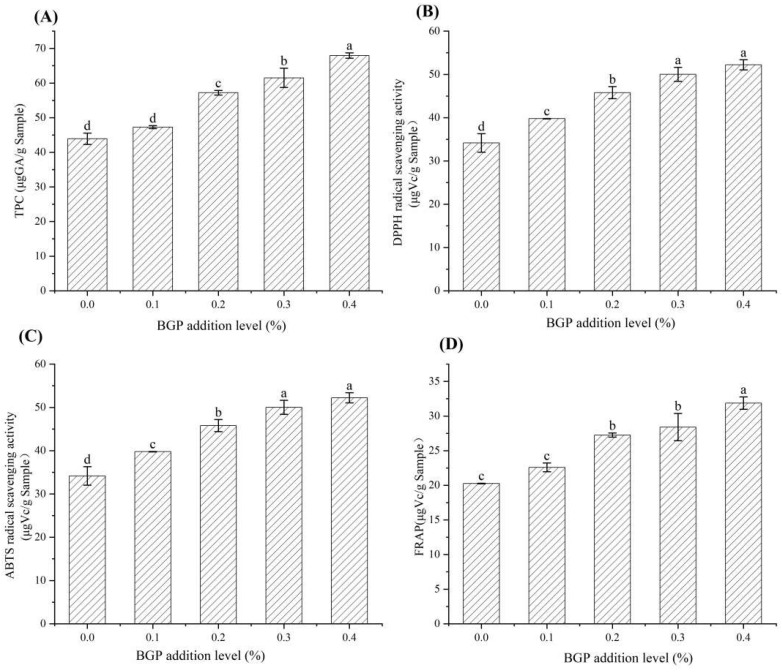
The antioxidant activity and TPC of yogurt with different levels of BGP. Different letters indicate significant differences (*p* < 0.05). (**A**) ABTS, (**B**) FRAP and (**C**) DPPH radical scavenging activity; (**D**) TPC (*n* = 3).

The TPC of yogurt with different levels of BGP is shown in Figure 5. As shown in Figure 5A, the TPC of the yogurt with 0.2%, 0.3% and 0.4% addition levels of BGP significantly increased (*p* < 0.05) by 30.28%, 40.05% and 54.74%, respectively, compared to the yogurt without BGP addition. Shori et al. found that cardamom and pepper extracts could increase the TPC in yogurt [28]. Yogurts with the addition of different fruit juices showed increased TPC due to the phenolic acids in the fruit juice [29]. Honey also increased the polyphenol content and antioxidant activity of a plant-based yogurt [30].

**Figure 6:**
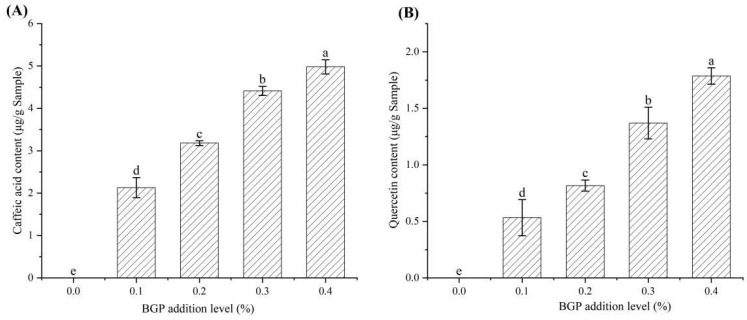
The content of quercetin (**A**) and caffeic acid (**B**) in yogurt with different levels of BGP. Different letters indicate significant differences (*p* < 0.05) (*n* = 3).

The content of quercetin and caffeic acid in the yogurt with the addition of BGP is shown in Figure 6. Quercetin and caffeic acid were not detected in the yogurt without BGP addition. The content of quercetin and caffeic acid in the yogurt significantly increased (*p* < 0.05) with increasing BGP addition. The content of quercetin and caffeic acid in the yogurt with the addition of 0.1–0.4% BGP was 0.53–1.79 mg/g and 2.13–4.98 mg/g, respectively. The content of quercetin and caffeic acid in the yogurt with the addition of 0.4% BGP was 3.35 and 2.34 times that of the yogurt with 0.1% BGP addition, respectively. It was also reported that *Rhus coriaria* leaf powder increased the gallic acid, vanillic acid and rosmarinic acid content of yogurt [31]. The quercetin and caffeic acid changes in the yogurt were similar to the TPC tendency of the yogurt. Therefore, both quercetin and caffeic acid were key functional components contributing to the antioxidant properties of yogurt.

### 2.7. Antioxidant Activity of Yogurt

The effects of BGP addition on the antioxidant activity of yogurt are shown in Figure 5. As shown in Figure 5, when the addition level of BGP in the yogurt was lower than 0.4%, the ABTS and DPPH radical scavenging capacities of the yogurt significantly increased (*p* < 0.05) with the increasing addition of BGP. The ABTS and DPPH radical scavenging activity of the yogurt with 0.3% and 0.4% levels of BGP showed no significant differences. The ABTS, FRAP and DPPH radical scavenging activity of yogurt with a 0.4% level of BGP increased by 52.79%, 57.31% and 42.51%, respectively, compared to the yogurt without BGP addition. The yogurt without BGP addition also exhibited low antioxidant activity, which was likely due to vitamins, free amino acids and bioactive peptides produced during fermentation [32]. Anuyahong et al. [33] reported that red rice rich in polyphenols and anthocyanins enhanced the antioxidant capacity of yogurt in a dose-dependent manner. These findings indicate that BGP significantly enhances the antioxidant properties and health benefits of yogurt.

### 2.8. Correlation Analysis

**Figure 7:**
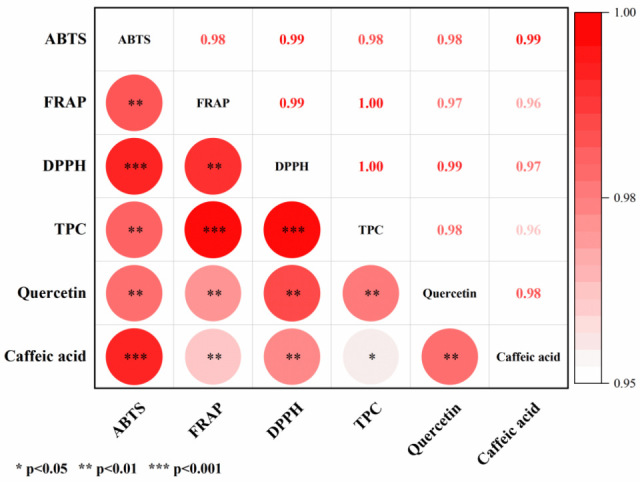
Correlation analysis between antioxidant capacity and polyphenol content of yogurt with different levels of BGP (*n* = 3). The deeper color indicates a higher correlation coefficient.

The correlation coefficients between the antioxidant activity, TPC and phenolic monomers are shown in Figure 7. All correlation coefficients between different indicators were above 0.95, suggesting that there were significant positive correlations (*p* < 0.05) between the phenolic acid content and antioxidant activity of yogurt with different levels of BGP. Therefore, both caffeic acid and quercetin in BGP contributed to the FRAP, ABTS and DPPH radical scavenging activity of yogurt. Similar results also showed that there was a strong correlation between TPC and antioxidant activity of yogurt fortified with apple pomace flour [34].

## 3. Conclusions

This study developed a kind of functional yogurt fortified with BGP. Although the addition of BGP significantly reduced the sensory scores of yogurt, the sensory scores of yogurt with 0.2% and 0.3% levels of BGP addition exceeded 80 points. The WHC and pH of yogurt were decreased with the increasing addition of BGP. The storage modulus (G’) and loss modulus (G”) of the yogurt with different levels of BGP increased with increasing shear frequencies. BGP addition promoted the formation of a more stable gel structure in the yogurt. Additionally, BGP addition significantly decreased the gel strength, rupture strength and hardness of the yogurt. The correlation analysis revealed that the antioxidant activity of yogurt had a significant and positive relationship with the phenolic acid content. Therefore, BGP could be used as a potential antioxidant ingredient to develop novel yogurts with high nutrition value. However, the effects of the storage time on the physicochemical characteristics and antioxidant activity of yogurt with BGP have been not investigated in this study. Future research should focus on the storage stability, probiotic properties and intestinal function regulation of yogurt with BGP.

## 4. Materials and Methods

### 4.1. Materials

Milk was purchased from the Inner Mongolia Mengniu Dairy (Group) by Share Ltd. (Hohhot, China), and the starter culture of *Lactobacillus bulgaricus* and *Streptococcus thermophilus* was obtained from Beijing Chuanxiu Technology Co., Ltd. (Beijing, China). The BGP extracts (purity: 10%) were supplied by Shanxi Kangyue Biotechnology Co., Ltd. (Xi’an, China). 1,1-Diphenyl-2-picrylhydrazyl (DPPH), 2,4,6-tripyridyl-s-triazine (TPTZ) and 2,2′-azino-bis(3-ethylbenzothiazoline-6-sulfonic acid) diammonium salt (ABTS) were all purchased from Shanghai Yuanye Biotechnology Co., Ltd. (Shanghai, China). Quercetin of HPLC grade and caffeic acid of HPLC grade were obtained from Chengdu Purify Technology Development Co., Ltd. (Chengdu, China). The remaining reagents were analytical reagents.

### 4.2. Preparation of BGP Extracts and Yogurt with BGP Addition

#### 4.2.1. Preparation of BGP Extracts

Crushed black garlic (100 g) was extracted in 80% ethanol (500 mL) in a sonicator for 30 min, and the filtered liquid was concentrated and purified through solid-phase extraction to obtain black garlic polyphenols. Finally, the BGP extract was obtained by spray drying.

#### 4.2.2. Preparation of Yogurt with BGP Addition

According to the method reported by Moreno-Ortega et al. [35], BGP at the BGP/milk mass ratios of 0%, 0.1%, 0.2%, 0.3% and 0.4% was supplemented with milk. Then, sugar at 8% was further mixed with the milk added with BGP. The above mixed milk was sterilized at 90 °C for 10 min. When the sterilized milk was cooled to 42 °C, 0.2% of the composite starter culture was added to the mixed milk and homogeneously mixed. The above milk was equally transferred into a 100 mL glass bottle and placed in a constant-temperature incubator at 42 °C for 6.5 h. Finally, the fermented yogurt was rapidly cooled to 4 °C in a refrigerator for 12 h to obtain the yogurt product. The yogurt with different levels of BGP was prepared in triplicate.

### 4.3. Sensory Evaluation

According to the sensory evaluation method reported by Rashwan et al., with some modifications [3], 12 assessors (six males and six females) majoring in food science, who had received training in sensory evaluation, were randomly selected to form a group of yogurt sensory evaluators. The sensory standard was developed based on the characteristics of yogurt with BGP addition. Each member independently scored different yogurt products without discussion. To facilitate an objective sensory evaluation, the yogurt was presented to panelists in plastic containers labeled with random codes. Panelists were instructed to cleanse their palates with filtered water between sample assessments to prevent cross-contamination of flavors and ensure the accurate evaluation of each formulation. The overall sensory evaluation (100 points) of the yogurt included taste (20 points), flavor (40 points), texture (30 points) and color (10 points).

### 4.4. WHC Measurement

According to the method reported by Du et al. [25], a 10 mL centrifuge tube was weighed and recorded as *m*_1_. Approximately 2.5 g of each yogurt was added into the tube, and the total mass of the centrifuge tube and yogurt was named *m*_2_. The yogurt was centrifuged at 10,000 g/min for 20 min at 25 °C. After centrifugation, the tube was allowed to stand for 5 min, and the supernatant was carefully removed. The mass of the centrifuge tube and precipitate was recorded as *m*_3_. The *WHC* of the yogurt was calculated according to the following Equation (1), performed in triplicate:

(1) $$WHC\left(\%\right)=\frac{m_{3}-m_{2}}{m_{2}-m_{1}}\times 100$$

### 4.5. Titratable Acidity Measurement

According to the method reported by Wang et al. [36], 5 g (*m*_0_) of yogurt was mixed with 20 mL of distilled water in a 100 mL conical flask, and the mixture was ultrasonicated at 400 W for 3 min (pulse duration of on-time 3 s and off-time 2 s). After the phenolphthalein indicator was added to the above solution, the mixture was titrated with 0.1 mol/L NaOH solution until the reaction solution remained red for 30 s. The volume of the NaOH solution (*V*_1_) was recorded. The titratable acidity of the yogurt was calculated according to the following Equation (2) and measured in triplicate:

(2) $$Titratable\;Acidity({}^{\circ}T)=\frac{V_{1}\times 100}{m_{0}}$$

### 4.6. pH Measurement

The yogurt with different addition levels of BGP was firstly homogenized using a homogenizer to ensure texture uniformity, and the pH value of the yogurt was measured by a calibrated PHS-3C pH meter (Shanghai Yueping Scientific Instrument Co., Ltd., Shanghai, China) in triplicate.

### 4.7. Rheological Property Measurement

The rheological properties of yogurt with different addition levels of BGP were measured by a TA DHR-2 rheometer (Waters Corporation, New Castle, DE, USA) in triplicate, following the method from Liu et al., with minor modifications [25]. After calibration, a 40-mm-diameter fixture was selected to measure the rheological properties of the yogurt. Dynamic frequency scanning was performed at 20 °C, with strain of 0.5% and a frequency range of 0.1–10 Hz.

### 4.8. Texture Measurement

The texture properties of yogurt with different addition levels of BGP were measured using a TA.XT Express texture analyzer (Stable Micro Systems, Ltd., Godalming, UK) in triplicate, according to the method of Najgebauer-Lejko et al., with minor modifications [37]. The parameters were as follows: a probe of P/0.5, a pre-test distance of 15 mm, a pre-test speed of 1.5 mm/s, a test speed of 1 mm/s, a post-test speed of 1 mm/s and a minimum trigger force of 5.0 g.

### 4.9. Extraction of Phenolic Acids in Yogurt with BGP Addition

Following the polyphenol extraction method reported by Khateeb et al., with some modifications [38], approximately 3.0 g of yogurt with BGP addition was weighed and placed into a 10 mL centrifuge tube, and 2 mL of anhydrous ethanol was further added. After the mixture was centrifuged at 8500 g/min for 20 min at 25 °C, the supernatant containing phenolic compounds was transferred to a 4 mL centrifuge tube for the determination of the antioxidant activity, TPC, quercetin and caffeic acid content.

#### 4.9.1. Total Phenolic Acid (TPC) Determination

The TPC in yogurt was determined according to the method reported by Cao et al., with minor modifications [39]. The 100 μL yogurt ethanol extract (100 μL) was firstly mixed with 125 μL of Folin–Ciocalteu reagent and 375 μL of 20% Na_2_CO_3_ solution in a 2 mL centrifuge tube, and 1400 μL distilled water was further added to the above mixture. The above mixed solution was allowed to react in the dark for 0.5 h. The absorbance value was measured at 765 nm in triplicate. A standard curve (y = 0.0054x + 0.0596, *R*^2^ = 0.9967) was plotted using gallic acid (GA) standard solutions (25, 50, 75, 100, 150, 200 μg/mL), and the TPC of yogurt was expressed as μg GA/g sample.

#### 4.9.2. UPLC Analysis of Quercetin and Caffeic Acid

According to the method reported by Simonetti et al. [31], a BEH C_18_ column (50 mm × 2.1 mm, 1.7 μm) equipped with a Waters UPLC system was used to determine the phenolic acids in yogurt at the detection wavelength of 280 nm, with a column temperature of 25 °C. Ultrapure water (A) and methanol (C) were selected as the mobile phases. The gradient elution program was as follows: 0–3 min, 10% C; 3–6 min, 80% C; 6–10 min, 80% C; 10–15 min, 10% C; 15–18 min, 10% C. The flow rate of the mobile phase was set at 0.2 mL/min with an injection volume of 8 μL. The quercetin standard curve of y = 5668.5x − 3833.7 (*R*^2^ = 0.999) and the caffeic acid standard curve of y = 1935.4x − 906.8 (*R*^2^ = 0.999) were used to calculate the content of quercetin and caffeic acid in the yogurt. The content of quercetin and caffeic acid was determined in triplicate and expressed as mg/g sample.

### 4.10. Antioxidant Activity Determination

#### 4.10.1. DPPH Radical Scavenging Activity Measurement

According to the method reported by Zadeh et al. [40], with some modifications, 100 μL yogurt extracts were mixed with 900 μL DPPH solution in a 2 mL centrifuge tube in the dark for 20 min. The absorption value of the reaction mixture was measured at 517 nm. A standard curve (y = 0.0152x − 0.036, *R*^2^ = 0.9855) was plotted using V_C_ standard solutions (10, 20, 30, 40, 50 μg/mL) as the abscissa and absorption values as the ordinate, and the DPPH radical scavenging activity of yogurt was expressed as μg V_C_/g sample in triplicate.

#### 4.10.2. ABTS Radical Scavenging Activity Measurement

According to the method reported by Gowd et al. [41], with some modifications, the absorbance value of the prepared ABTS solution at 734 nm was near 1.0. A standard curve (y = 0.0152x − 0.0461, *R*^2^ = 0.9958) was plotted using V_C_ standard solutions (5, 10, 20, 30, 40, 50 μg/mL), and the ABTS radical scavenging activity of different yogurts was expressed as μg V_C_/g sample in triplicate.

#### 4.10.3. Ferric Reducing Antioxidant Power (FRAP) Measurement

The FRAP solution was prepared according to Gowd et al. [41]. Then, 100 μL yogurt extract and 900 μL FRAP solution were mixed in a 2 mL centrifuge tube in the dark for 30 min. The absorption value of the mixture was measured at 593 nm in triplicate. A standard curve (y = 0.0211x − 0.0278, *R*^2^ = 0.9981) was plotted using V_C_ standard solutions (5, 10, 20, 30, 50 μg/mL), and the FRAP was expressed as μg V_C_/g sample.

### 4.11. Data Analysis

All experiments were performed in triplicate, and the results were expressed as the mean ± SD. The statistical analysis was conducted using SPSS 25.0 with ANOVA. The level of statistical significance was set at *p* < 0.05.

## Figures and Tables

**Table 1:** The sensory evaluation of yogurt with different levels of BGP.

BGP Addition Level	Flavor (40)	Texture (30)	Taste (20)	Color (10)	Total Sensory Score (100)
0%	36.67 ± 0.52 ^a^	27.50 ± 0.55 ^a^	18.33 ± 0.52 ^a^	8.50 ± 0.55 ^a^	91.00 ± 1.41 ^a^
0.1%	31.50 ± 0.84 ^c^	25.00 ± 0.89 ^b^	15.17 ± 1.17 ^b^	7.83 ± 0.41 ^a^	79.50 ± 1.76 ^c^
0.2%	33.67 ± 0.82 ^b^	25.00 ± 0.63 ^b^	15.83 ± 0.41 ^b^	7.83 ± 0.41 ^a^	82.33 ± 0.82 ^b^
0.3%	34.50 ± 0.55 ^b^	25.33 ± 0.52 ^b^	16.17 ± 1.83 ^b^	8.00 ± 0.89 ^a^	84.00 ± 2.28 ^b^
0.4%	29.56 ± 2.19 ^d^	22.83 ± 1.17 ^c^	11.33 ± 1.21 ^c^	6.67 ± 0.52 ^b^	70.67 ± 1.75 ^d^

Different lowercase superscript letters in the same column indicate significant differences (*p* < 0.05). (*n* = 12).

**Table 2:** The texture characteristics of yogurt with different levels of BGP.

BGP Addition Level	Gel Strength /g	Rupture Strength/g	Hardness /g	Chewiness /g·s	Stickiness /g·s
0%	18.56 ± 0.06 ^a^	25.65 ± 0.30 ^b^	25.65 ± 0.30 ^b^	318.88 ± 2.42 ^b^	−79.53 ± 1.85 ^b^
0.1%	18.96 ± 0.24 ^a^	27.69 ± 0.20 ^a^	27.69 ± 0.20 ^a^	336.14 ± 4.86 ^a^	−85.07 ± 1.32 ^a^
0.2%	17.84 ± 0.01 ^b^	24.96 ± 0.16 ^c^	24.96 ± 0.16 ^c^	313.3 ± 6.35 ^b^	−78.93 ± 0.10 ^b^
0.3%	17.25 ± 0.04 ^c^	24.95 ± 0.28 ^c^	24.95 ± 0.28 ^c^	306.14 ± 6.29 ^b^	−80.06 ± 0.00 ^b^
0.4%	16.09 ± 0.39 ^d^	23.49 ± 0.08 ^d^	23.49 ± 0.08 ^d^	284.24 ± 4.89 ^c^	−78.78 ± 0.00 ^b^

Different lowercase superscript letters in the same column indicate significant differences (*p* < 0.05) (*n* = 3).

## Data Availability

The original contributions presented in this study are included in the article. Further inquiries can be directed to the corresponding author.
